# Identification of Penexanthone A as a Novel Chemosensitizer to Induce Ferroptosis by Targeting Nrf2 in Human Colorectal Cancer Cells

**DOI:** 10.3390/md22080357

**Published:** 2024-08-06

**Authors:** Genshi Zhao, Yanying Liu, Xia Wei, Chunxia Yang, Junfei Lu, Shihuan Yan, Xiaolin Ma, Xue Cheng, Zhengliang You, Yue Ding, Hongwei Guo, Zhiheng Su, Shangping Xing, Dan Zhu

**Affiliations:** 1 First Clinical Medical College, Guangxi Medical University, Nanning 530021, China; 202120322@sr.gxmu.edu.cn (G.Z.); 202220339@sr.gxmu.edu.cn (X.C.); 2 Pharmaceutical College, Guangxi Medical University, Nanning 530021, China; liuyanying@sr.gxmu.edu.cn (Y.L.); weixia@gxykdx1.wecom.work (X.W.); 202121056@sr.gxmu.edu.cn (C.Y.); ljf@sr.gxmu.edu.cn (J.L.); 201921075@sr.gxmu.edu.cn (S.Y.); 202121038@sr.gxmu.edu.cn (X.M.); 202221159@sr.gxmu.edu.cn (Z.Y.); 202321034@sr.gxmu.edu.cn (Y.D.); hongweiguo@gxmu.edu.cn (H.G.); suzhiheng@gxmu.edu.cn (Z.S.); 3 Guangxi Key Laboratory for Bioactive Molecules Research and Evaluation, Nanning 530021, China

**Keywords:** penexanthone A, ferroptosis, Nrf2, cisplatin, colorectal cancer

## Abstract

Ferroptosis has emerged as a potential mechanism for enhancing the efficacy of chemotherapy in cancer treatment. By suppressing nuclear factor erythroid 2-related factor 2 (Nrf2), cancer cells may lose their ability to counteract the oxidative stress induced by chemotherapy, thereby becoming more susceptible to ferroptosis. In this study, we investigate the potential of penexanthone A (PXA), a xanthone dimer component derived from the endophytic fungus *Diaporthe goulteri,* obtained from mangrove plant *Acanthus ilicifolius*, to enhance the therapeutic effect of cisplatin (CDDP) on colorectal cancer (CRC) by inhibiting Nrf2. The present study reported that PXA significantly improved the ability of CDDP to inhibit the activity of and induce apoptosis in CRC cells. Moreover, PXA was found to increase the level of oxidative stress and DNA damage caused by CDDP. In addition, the overexpression of Nrf2 reversed the DNA damage and ferroptosis induced by the combination of PXA and CDDP. In vivo experiments using zebrafish xenograft models demonstrated that PXA enhanced the therapeutic effect of CDDP on CRC. These studies suggest that PXA enhanced the sensitivity of CRC to CDDP and induce ferroptosis by targeting Nrf2 inhibition, indicating that PXA might serve as a novel anticancer drug in combination chemotherapy.

## 1. Introduction

Colorectal cancer (CRC) stands as the most prevalent form of malignant tumor within the digestive tract and ranks as the second-leading cause of cancer-related mortality globally [1]. Cisplatin (CDDP), a DNA-damaging chemotherapeutic agent, has been approved for treating various solid tumors, including CRC [2]. Nevertheless, the frequent development of intrinsic and acquired chemoresistance hinders its therapeutic efficacy [3]. Consequently, there is an ongoing need for effective strategies to enhance CDDP sensitivity in CRC treatment.

Ferroptosis, an iron-dependent form of regulated cell death, occurs when lipid peroxides accumulate to lethal levels on cellular membranes [4]. This process is distinct from apoptosis and involves different cellular mechanisms, making it a compelling target for combination therapy with CDDP [5]. Several studies have reported that CDDP can synergize with ferroptosis inducers, such as Erastin and RSL3, in multiple cancer types [6,7,8]. Therefore, ferroptosis presents a novel therapeutic method for enhancing the efficacy of CDDP in CRC treatment [9]. As a master regulator of antioxidant defense, the transcription regulator nuclear factor erythroid 2-related factor 2 (Nrf2) governs the transcription of numerous genes implicated in glutathione peroxidase 4 (GPX4)-glutathione (GSH)-associated ferroptosis defenses, including solute carrier family 7 member 11 (SLC7A11) [10]. This regulatory function mitigates the vulnerability of cancer cells to ferroptosis. Furthermore, Nrf2 is overexpressed in diverse human malignancies, and its downstream targets are intricately associated with the suppression of the ferroptotic cascade [11]. It has been reported that various natural products can induce ferroptosis to enhance the sensitivity of CDDP to cancer cells by inhibiting the Nrf2 pathway [10,12,13]. Notably, brusatol, a potent Nrf2 inhibitor, sensitizes esophageal adenocarcinoma cells to CDDP by inducing ferroptosis [14]. Similarly, ginkgetin, a natural anti-cancer product, also promotes CDDP-induced anticancer effects and triggers ferroptosis in lung cancer cells by inhibiting the Nrf2 pathway [15]. Consequently, targeting the Nrf2 pathway to induce ferroptosis can enhance the therapeutic efficacy of CDDP.

Marine fungi are known for their unique metabolic pathways that are different from those of terrestrial fungi, making them a potential source of novel bioactive compounds [16]. *Acanthus ilicifolius* L. is an important medicinal plant in mangrove forests, which is rich in secondary metabolites with various biological activities [17]. It is of great research value to search for lead molecules with significant anti-tumor activity from marine fungi. In this study, we incidentally isolated penexanthone A (PXA), a xanthone dimer, from the metabolites of the fungus *Diaporthe goulteri* collected from *Acanthus ilicifolius* L. It has been reported that PXA can also be isolated from other fungus, such as *Phomopsis* sp. [18] and *Penicillium* sp. [19]. However, the anticancer potential and underlying molecular mechanisms of PXA have not yet been explored, which was thus the aim of this study. Here, we investigated whether PXA enhances the sensitivity of CRC cells to CDDP-induced ferroptosis by inhibiting the Nrf2 signaling pathway. Our results demonstrated that PXA possesses interesting chemosensitizing properties, and the combination of PXA and CDDP may provide a novel therapeutic strategy for CRC.

## 2. Results

### 2.1. Structural Identification of PXA

**Figure 1:**
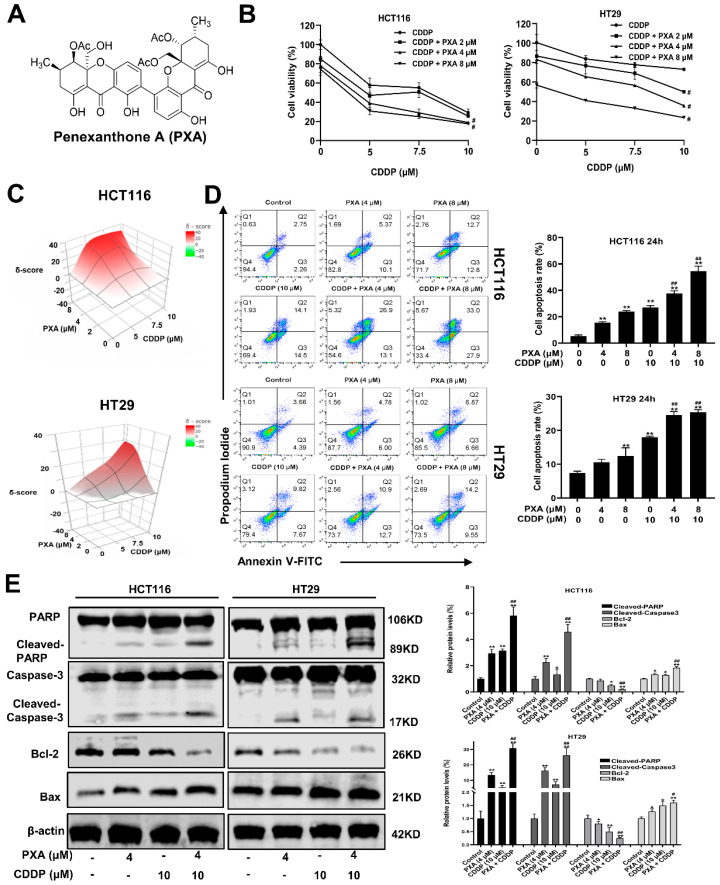
PXA sensitizes CRC cells to CDDP-induced cytotoxicity and apoptosis. (**A**) Chemical structural of PXA. (**B**) CRC cells were co-treated with PXA and CDDP for 48 h, and the percentage of cell viability was determined by CCK-8 assay. (**C**) 3D visualization of synergy scores between PXA and CDDP obtained using the SynergyFinder tool; these calculated average synergy scores are 20.7 and 11.3 for these two panels of drug combinations (Synergy scores > 10 are considered synergistic). (**D**) The percentage of apoptotic cells was analyzed and quantified using flow cytometry after Annexin V-FITC/PI staining. (**E**) The protein levels of Cleaved-PARP, Cleaved-caspase-3, BAX, and Bcl-2 in HCT116 and HT29 cells were detected by Western blot after 24 h treatment; β-acting was used as a loading control. Results are expressed as means ± SD. * *p* < 0.05, ** *p* < 0.01 versus the control group, ^#^ *p* < 0.05, ^##^ *p* < 0.01 versus the CDDP-treatment group.

The compound isolated from the metabolites of the fungus *Diaporthe goulteri* was obtained as a yellow amorphous powder and characterized with UV, IR, ^1^H NMR, ^13^C NMR and MS spectra (Figures S1–S5). The purity of PXA was greater than 99%, as determined by HPLC (Figure S6). [α]^25^_D_ −16.2 (c 0.1, MeOH); UV (MeOH)λ_max_ (log ε) 338 (4.25) nm; IR (KBr) ν_max_ 3490, 2967, 2933, 2360, 1749, 1611, 1564, 1441, 1368, 1322, 1222, 1135, 1096, 1047, 990, 883, 818, 738, 660, 600, 539, 485 cm^−1^. Analysis of ^1^H NMR and ^13^C NMR spectra was as follows: ^1^H NMR (600 MHz, CDCl_3_) δ 7.35 (d, *J* = 8.0 Hz, 1H), 7.20 (d, *J* = 8.0 Hz, 1H), 6.58 (d, *J* = 8.0 Hz, 1H), 6.44 (d, *J* = 8.0 Hz, 1H), 5.61 (br s, 1H), 5.34 (br s, 1H), 4.57 (d, *J* = 13.8 Hz, 2H), 3.99 (d, *J* = 14.3 Hz, 2H), 3.90 (d, *J* = 13.8 Hz, 2H), 3.76 (d, *J* = 14.3 Hz, 2H), 2.50 (m, 2H), 2.50 (m, 2H), 2.40 (m, 1H), 2.40 (m, 1H), 2.15 (s, 3H), 2.10 (s, 3H), 1.87 (s, 3H), 1.10 (d, *J* = 7.0 Hz, 3H), 1.00 (d, *J* = 7.0 Hz, 3H); ^13^C NMR (150 MHz, CDCl_3_) δ: 188.0, 188.0, 178.1, 177.4, 170.7, 170.7, 170.0, 161.5, 159.8, 157.4, 154.5, 139.0, 138.7, 118.2, 117.1, 109.9, 107.3, 106.5, 106.5, 101.2, 100.2, 82.4, 80.0, 71.7, 69.4, 65.4, 63.8, 33.4, 33.0, 27.5, 27.5, 21.0, 20.9, 20.5, 17.5, 17.2; HRMS (ESI): *m*/*z* = 709.2130 [M + H]^+^, calculated for C_36_H_37_O_15_, 709.2132, confirming the molecular formula as C_36_H_36_O_15_. A comparison of the compound spectral data with the literature data [19] proved it to be PXA (Figure 1A).

### 2.2. Effect of PXA on CDDP-Induced Cytotoxicity and Apoptosis in CRC Cells

To determine the potential application of PXA in the treatment of certain cancers, we first investigated the cytotoxicity of PXA to five cancer cell lines. As shown in Table S1, PXA exhibited the best inhibitory effect on HCT116 cells compared to other cancer cell lines (SKOV3, MCF-7, A549, and PC3). To further explore the therapeutic application of PXA in CRC cells, we assessed whether PXA exerts chemosensitizing activity. Low-concentration PXA was used to test its synergistic effects with CDDP using cell viability assays and SynergyFinder software (Release (3.19)). Interestingly, PXA showed obvious synergistic effects with CDDP in reducing the cell viability of CRC (*p* < 0.05, Figure 1B), with calculated synergy scores being > 10 (Figure 1C). Flow cytometry analysis revealed that PXA (4 and 8 μM) significantly reinforced CDDP-induced apoptosis in both HCT116 and HT29 cells (*p* < 0.05, Figure 1D). Consistently, Western blotting further found that the co-treatment of PXA and CDDP in HCT116 and HT29 cells led to a reduced expression of Bcl-2, the activation of Bax, and the cleavage activation of caspase-3 and PARP, compared with that caused by CDDP-alone (*p* < 0.05, Figure 1E). These results indicated that PXA could potentiate CDDP-induced CRC cell apoptosis effectively and act as an adjuvant with CDDP.

### 2.3. Effect of PXA on CDDP-Induced ROS in CRC Cells

**Figure 2:**
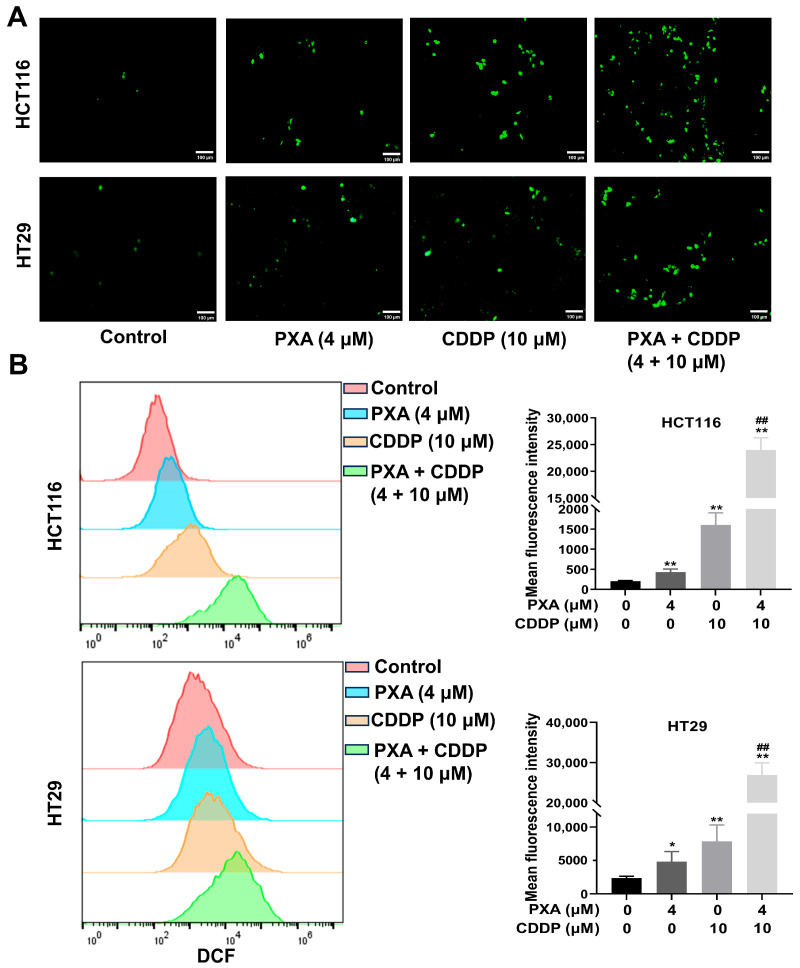
PXA increased CDDP-induced ROS production. (**A**,**B**) HCT116 and HT29 cells treated with PXA and CDDP were incubated with the DCFH-DA probe for 20 min, and the ROS levels (DCF fluorescence) were observed and analyzed by fluorescence microscopy (**A**) and flow cytometry (**B**). Scale bars: 100 μm. Results are expressed as means ± SD. * *p* < 0.05, ** *p* < 0.01 versus the control group, ^##^ *p* < 0.01 versus the CDDP-treatment group.

The effects of PXA and CDDP on the cellular ROS levels in HCT116 and HT29 cells were analyzed with the DCFH-DA probe kit. As shown in Figure 2A, the combination of PXA and CDDP significantly increased the levels of cellular ROS in HCT116 and HT29 cells, as indicated by an increase in the DCF-liberated fluorescent signal. Meanwhile, flow cytometry confirmed that PXA or CDDP treatment increased the cellular ROS levels, and these ROS signals were significantly increased by the combination treatment of PXA and CDDP in HCT116 and HT29 cells (*p* < 0.05, Figure 2B). These results suggested that PXA enhanced the sensitivity of CRC cells to CDDP, which is associated with ROS generation.

### 2.4. Effect of PXA on CDDP-Induced DNA Damage and Oxidative Stress in CRC Cells

**Figure 3:**
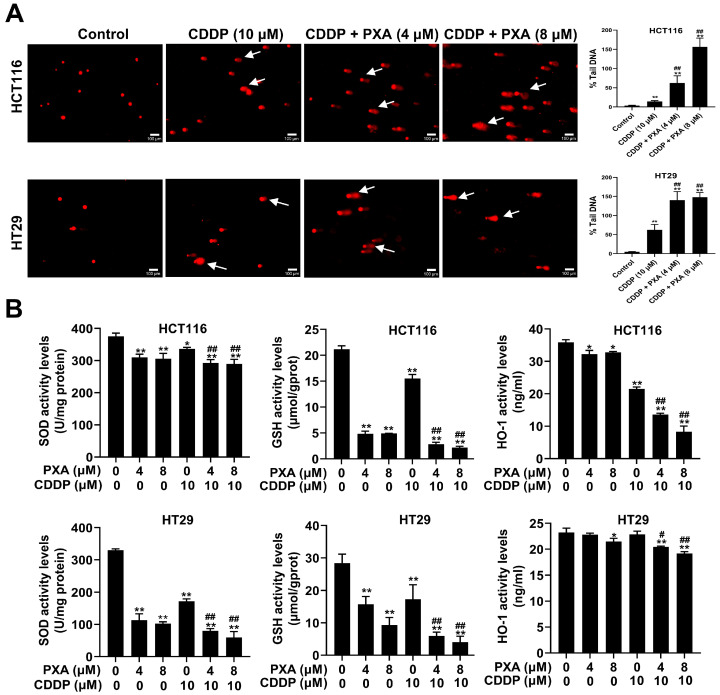
PXA increased CDDP-induced DNA damage and oxidative stress. (**A**) DNA damage levels in HCT116 and HT29 cells treated with PXA and CDDP for 24 h were assessed using the comet assay. Scale bars: 100 μm. (**B**) After 24 h of treating HCT116 and HT29 cells with PXA and CDDP, the content of GSH, SOD, and HO-1 was measured using ELISA. Results are expressed as means ± SD. * *p* < 0.05, ** *p* < 0.01 versus the control group, ^#^ *p* < 0.05, ^##^ *p* < 0.01 versus the CDDP-treatment group.

The accumulation of ROS can lead to oxidative stress, which can cause damage to cellular components, including DNA [20,21]. To assess the extent of DNA damage induced by PXA and CDDP, a comet assay was employed. The results from the comet assay indicated that the combination of PXA and CDDP significantly increased the extension of the comet tail length compared to treatment with CDDP alone (*p* < 0.05, Figure 3A). Biochemical kits were used to measure cellular antioxidants such as GSH, Superoxide Dismutase (SOD) and Heme Oxygenase-1 (HO-1). The results revealed that the levels of GSH, SOD, and HO-1 in the PXA and CDDP combination group were remarkably reduced compared with the group undergoing treatment with CDDP alone (*p* < 0.05, Figure 3B). These results demonstrated that the co-treatment of PXA and CDDP induced oxidative stress, leading to DNA damage in CRC cells, and PXA increased the sensitivity of CRC cells to CDDP-induced DNA damage.

### 2.5. Effect of PXA on Nrf2 Protein in CRC Cells

**Figure 4:**
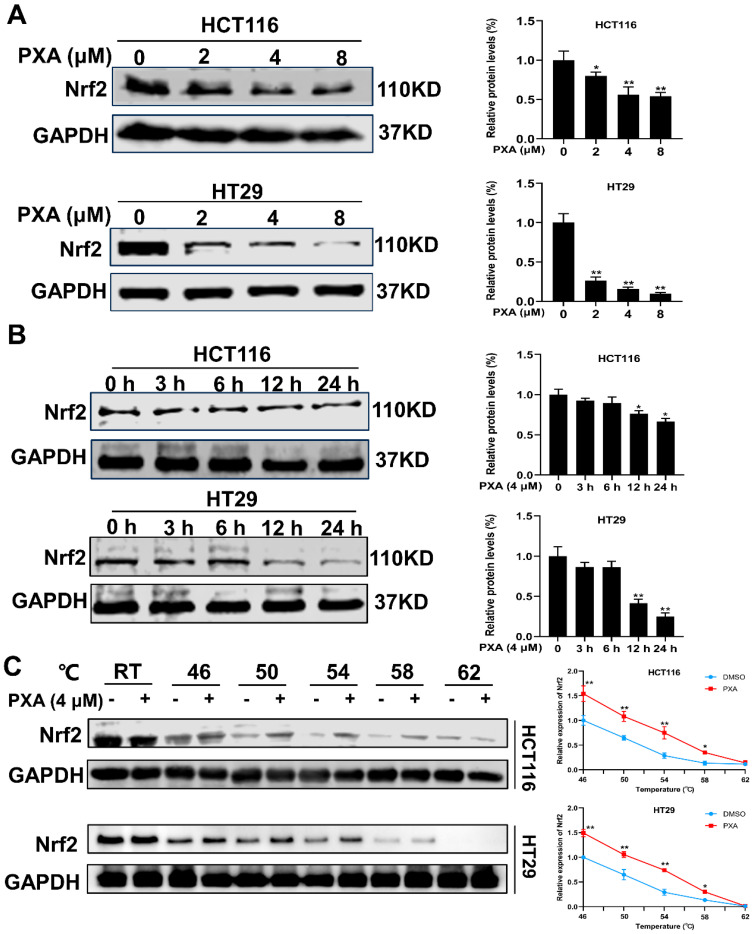
PXA inhibits Nrf2 protein expression. (**A**,**B**) Western blotting analyses of Nrf2 expression in HCT116 and HT29 cells treated with PXA for indicated concentrations (**A**) and time points (**B**). (**C**) CETSA was performed to confirm that PXA targets Nrf2 proteins. Results are expressed as means ± SD. * *p* < 0.05, ** *p* < 0.01 versus the control group.

Nrf2 is a key transcription factor known for its role in the cellular defense against oxidative stress and plays a crucial role in preventing lipid peroxidation and ferroptosis [10]. PXA was found to reduce the level of Nrf2 protein expression in a dose- and time-dependent manner in HCT116 and HT29 cells (*p* < 0.05, Figure 4A,B). To further investigate the interaction between PXA and Nrf2 protein, a CETSA was performed on CRC cells. The CETSA results indicated an increase in Nrf2 expression after PXA treatment compared to controls under various high-temperature conditions (Figure 4C). This suggests that PXA binding to the Nrf2 protein increases its thermal stability, making it less susceptible to denaturation and precipitation. These initial findings suggested that PXA could potentiate the inhibitory effect of CDDP on CRC cells by causing the degradation of the Nrf2 protein.

### 2.6. Overexpression of Nrf2 Reverses the Sensitization Effect of PXA on CDDP

**Figure 5:**
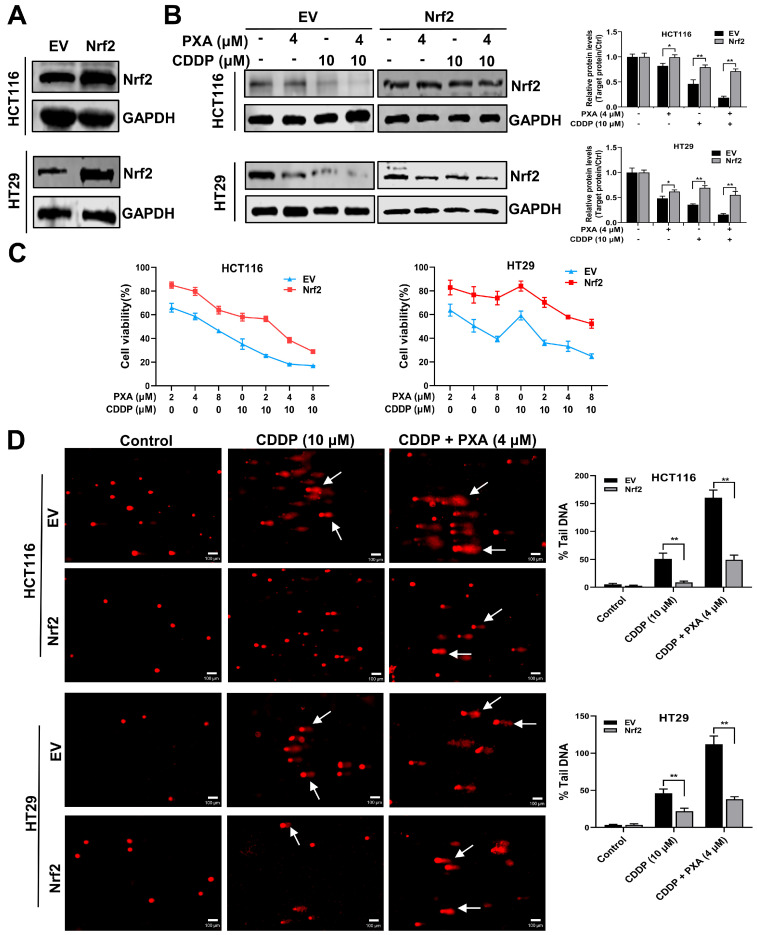
Nrf2 overexpression reverses the chemosensitizing activity of PXA. (**A**) Protein levels of Nrf2 in stably overexpressing empty vector (EV) and Nrf2 HCT116 and HT29 cells were examined by Western blotting. (**B**) Western blot assay to detect the effect of PXA in combination with CDDP on Nrf2 protein in EV and Nrf2 stable overexpressing HCT116 and HT29 cells. (**C**) CCK-8 assay was performed on EV and Nrf2 stable overexpressing HCT116 and HT29 cells treated with PXA and CDDP for 48 h. (**D**) The DNA damage levels of EV and Nrf2 stable overexpressing HCT116 and HT29 cells treated with PXA and CDDP for 24 h were evaluated using the comet assay. Scale bars: 100 μm. Results are expressed as means ± SD. * *p* < 0.05, ** *p* < 0.01 versus the EV group.

To verify whether PXA enhances the sensitivity of CRC cells to CDDP by targeting Nrf2, we established a stable overexpression of Nrf2 in HCT116 and HT29 cells (Figure 5A). The capacities of PXA combined with CDDP on reducing Nrf2 expression were significantly attenuated by Nrf2 overexpression (*p* < 0.05, Figure 5B). Consistently, Nrf2 overexpression partially reversed the inhibitory effects of PXA combined with CDDP on cell survival (Figure 5C). Moreover, Nrf2 overexpression also attenuated DNA damage caused by the combination of PXA and CDDP (Figure 5D). Therefore, these results demonstrated that PXA enhanced the sensitivity of CRC cells to CDDP by inhibiting Nrf2.

### 2.7. Effect of Nrf2 on PXA Enhanced CDDP-Induced Ferroptosis

**Figure 6:**
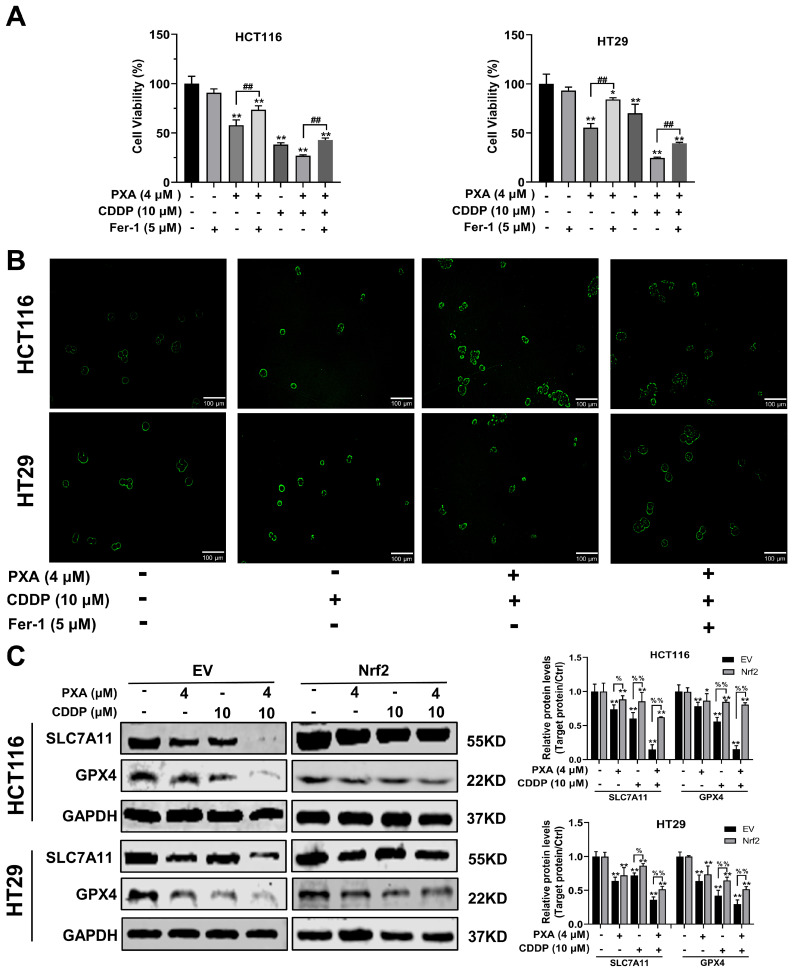
PXA enhances CDDP-induced ferroptosis by inhibiting Nrf2 pathway. (**A**) HCT116 and HT29 cells were treated with PXA and CDDP with or without ferroptosis inhibitor (Fer-1) for 24h, and cell viability was measured using the CCK-8 assay. (**B**) Detection of lipid hydroperoxides by fluorescence imaging of Liperfluo in HCT116 and HT29 cells treated with PXA and CDDP with or without Fer-1. Scale bars: 100 μm. (**C**) EV and Nrf2 stable overexpressing HCT116 and HT29 cells were treated with PXA and CDDP for 24 h and the expression of SLC7A11 and GPX4 were examined by Western blotting. Results are expressed as means ± SD. * *p* < 0.05, ** *p* < 0.01 versus the control group, ^##^ *p* < 0.01 versus the Fer-1 group, ^%^ *p* < 0.05, ^%%^ *p* < 0.01 versus the EV group.

To investigate whether PXA can enhance the sensitivity of CRC cells to CDDP by inducing ferroptosis, we conducted a series of experiments using the ferroptosis inhibitor Fer-1. Firstly, the viability of CRC cells was examined after treatment with PXA, both alone and in combination with CDDP, in the presence or absence of Fer-1. The results indicated that the inhibitory effect of PXA or its combination with CDDP on the viability of CRC cells was significantly reversed when Fer-1 was added to the treatment (*p* < 0.05, Figure 6A). In addition, Fer-1 was also able to inhibit the generation of lipid peroxides induced by the combination of PXA and CDDP, which further confirms the importance of ferroptosis in the mechanism of PXA action (Figure 6B). Western blot results showed that PXA significantly inhibited the expression of SLC7A11 and GPX4. Notably, the combination of PXA with CDDP resulted in significantly lower expression levels of SLC7A11 and GPX4 compared to either agent alone. However, Nrf2 overexpression could significantly alleviate the inhibitory effect of PXA or its combination with CDDP on SLC7A11 and GPX4 protein expression (*p* < 0.05, Figure 6C). These findings suggested that PXA induced ferroptosis in CRC cells by inhibiting the Nrf2 signaling pathway, thereby enhancing the sensitivity of CRC cells to CDDP.

### 2.8. Effect of PXA and CDDP on CRC Xenograft Zebrafish

**Figure 7:**
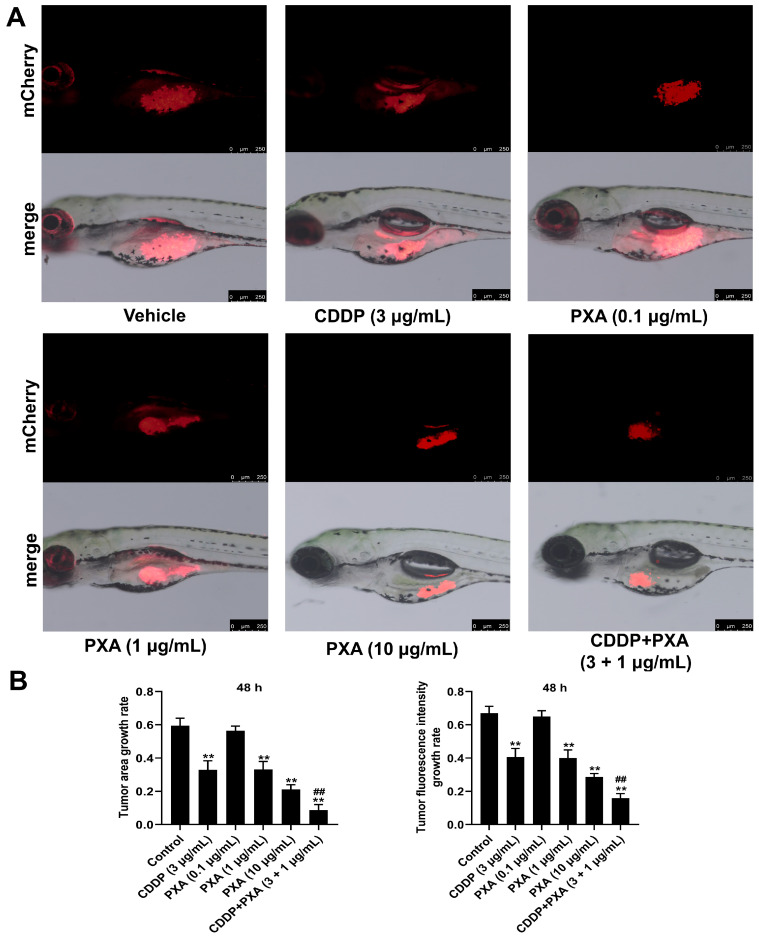
PXA enhances the therapeutic efficacy of CDDP in CRC xenograft zebrafish model. (**A**) HCT116-CM-Dil cells were injected into zebrafish embryo. At the end of the experiments, phenotypic map of fluorescence of HCT116-CM-Dil cells in zebrafish were photographed. Scale bars: 250 μm (**B**) The fluorescence area and intensity of HCT116-CM-Dil cells in zebrafish were analyzed by Image J software (Version 1.54j). Results are expressed as means ± SD. ** *p* < 0.01 versus the control group, *^##^ p* < 0.01 versus the CDDP-treatment group.

We further examined whether the combination of PXA and CDDP could serve as a promising therapeutic strategy using a CRC xenograft zebrafish model. HCT116 cells labeled with red, fluorescent dye (CM-Dil) were injected into the zebrafish embryos by a microinjector to establish the xenograft zebrafish model. After a 48 h treatment of PXA and CDDP, the fluorescence intensity of the zebrafish in each group was evaluated via a fluorescent stereo-microscope to assess the therapeutic potential of PXA in vivo. Our results showed that PXA (0.1 and 10 μg/mL) or CDDP (3 μg/mL) at relatively low doses moderately reduced the fluorescence intensity and area of the zebrafish; however, PXA and CDDP co-administration led to a more significant reduction in the fluorescence intensity and area of the zebrafish, compared with the CDDP treatment group (*p* < 0.05, Figure 7A,B). Therefore, the results revealed that PXA efficiently enhanced the sensitivity of CRC to CDDP in vivo.

## 3. Discussion

CDDP is an extensively utilized potent chemotherapeutic agent in various malignant tumors including CRC, but drug resistance persists as the primary challenge in clinical application [22]. Therefore, the development of innovative chemosensitizers with robust anti-cancer potency for CDDP is indispensable. The mechanisms by which the anti-tumor activity of CDDP is achieved have been studied extensively in various cancer cells, and there is strong evidence that CDDP exerts anticancer effects mainly by interacting with DNA resulting in DNA damage and activating the enzyme NADPH oxidase to trigger an excessive production of ROS [23,24]. In this study, we firstly found that co-treatment with PXA and CDDP produced synergistic cytotoxic effects on HCT116 and HT29 cells. Secondly, PXA markedly sensitized CRC cells to CDDP-induced apoptosis and DNA damage. CRC xenograft zebrafish studies also showed that the combination of PXA and CDDP enhanced the sensitivity of CRC to CDDP. Most notably, our results demonstrated that PXA in combination with CDDP promoted ROS accumulation, which was accompanied by the depletion of the antioxidants GSH, SOD, and HO-1. Ferroptosis is characterized by the depletion of intracellular GSH, the accumulation of ROS, and ultimately oxidative cell death [25]. It has been reported that ferroptosis inducers can significantly increase the sensitivity to chemotherapeutic drugs such as CDDP, which suggests that targeting ferroptosis can be considered a promising therapeutic strategy in CDDP-resistant cancer cells [26]. The balance between ROS production and antioxidant defenses is critical for determining the susceptibility to ferroptosis [27]. Given that the combination treatment of PXA and CDDP significantly promoted ROS accumulation and GSH depletion, we hypothesized that PXA enhanced the CDDP chemosensitivity of CRC by inducing ferroptosis.

Multiple studies have provided evidence that GPX4 and SLC7A11 are crucial regulators of ferroptosis, playing a critical role in mitigating cellular lipid peroxidation [28,29,30]. The inhibition of GPX4 and SLC7A11 can lead to GSH depletion and subsequently increase the sensitivity of cells to ferroptosis [31]. Our data showed that the incubation of CRC cells in the combination treatment of PXA and CDDP induced an increase in the fluorescence signal from Liperfluo compared to the CDDP group, indicating the accumulation of hydroperoxy-lipids. In addition, ferroptosis inhibitor (Fer-1) can reverse the CRC cell growth inhibition and lipid peroxidation caused by the combination of PXA and CDDP. Furthermore, the protein expression of GPX4 and SLC7A11 proteins was significantly decreased by PXA in combination with CDDP treatment, as compared with the CDDP group. Our results demonstrated that PXA increased the sensitivity of CRC cells to CDDP by enhancing ferroptosis.

**Figure 8:**
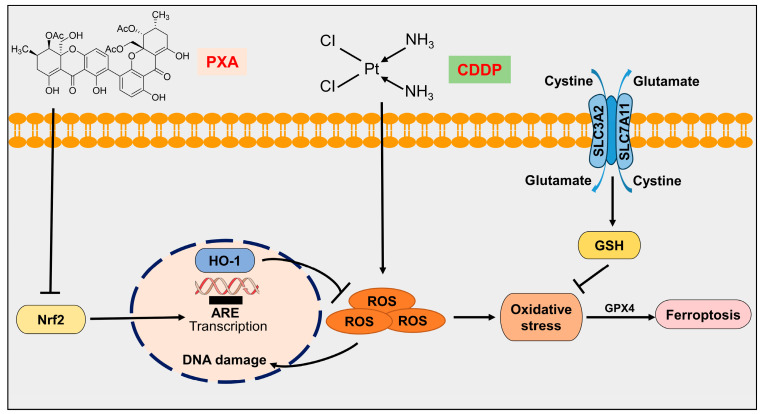
Schematic representation of PXA enhancing the sensitivity of CRC cells to CDDP by inducing ferroptosis through the inhibition of Nrf2. “↓” indicates promotion; “⊥” indicates inhibition.

Accumulating evidence has shown that Nrf2 pathway inhibitors are promising chemosensitizers [32,33]. Nrf2, as a master regulator of antioxidants, has been reported to control the regulatory pathways of ferroptosis, which involve proteins associated with GSH synthesis/metabolism (i.e., SLC7A11, GCL, GSS, GSTP1, and PRDX1), ferroptosis regulators (i.e., xCT, GPX4, FSP1, and GCH1), iron metabolism/ferritinophagy (i.e., FTH1, FTL, FPN, ATG5, ATG7 ULK1/2, and HMOX1), and lipid metabolism (i.e., ALOX12, POR, and NQO1) [10,11,34]. Alterations in the Nrf2 pathway are major events involved in redox homeostasis and ferroptosis [35]. Nrf2 overexpression in many types of cancers enhanced proliferation and contributed to chemoresistance [36]. Experimental evidence suggests that Nrf2 inhibitors are frequently used in combination with chemotherapeutic drugs to sensitize a broad spectrum of cancer cells to CDDP or other chemotherapeutic drugs by inducing ferroptosis [10,37,38,39]. Tiliroside, novel ferroptosis inducer, enhances the sensitivity of sorafenib to HepG2 and Hep3B cells by promoting Nrf2 degradation [40]. Withaferin A, a natural steroidal lactone extracted from *Withania somnifera*, sensitizes HepG2 cells to sorafenib via Nrf2-mediated ferroptosis [41]. Eicosapentaenoic acid, a polyunsaturated fatty acid, enhances the chemosensitivity of osteosarcoma to CDDP by inducing ferroptosis through the Nrf2 pathway [42]. Therefore, we tried to demonstrate whether PXA enhances the sensitivity of CRC cells to CDDP by inducing ferroptosis through targeting Nrf2. In our study, we confirmed Nrf2 as a potential target of PXA, and Nrf2 overexpression markedly reversed the inhibitory effect on CRC cell survival and the occurrence of ferroptosis induced by the combination of PXA and CDDP. These results indicate that PXA may be a promising chemosensitizer (Figure 8).

## 4. Materials and Methods

### 4.1. Reagents and Antibodies

CDDP and Fer-1 were purchased from MedChemExpress (Princeton, NJ, USA). Cell Count Kit-8 (CCK-8) was purchased from Meilunbio (Dalian, China). The protease inhibitor cocktail was purchased from Apexbio (Houston, TX, USA). Fetal bovine serum (FBS), RPMI-1640 and McCoy’s 5A medium were obtained from Gibco (Grand Island, NY, USA). The recombinant lentivirus targeting Nrf2 for humans was purchased from GenePharma (Suzhou, China). The RIPA buffer, ROS assay kit and BCA assay kit were purchased from Beyotime Biotechnology (Guangzhou, China). The Annexin V-FITC/PI apoptosis detection kit was purchased from Multi Sciences (Hangzhou, China). The kits used for the comet assay and GSH and SOD assays were obtained from Nanjing Jiancheng Bioengineering Institute (Nanjing, China). The HO-1 assay kit was purchased from Elabscience (Wuhan, China). The Liperfluo assay kit was purchased from Dojindo (Kumamoto, Japan). CM-Dil cell-labeling solution was purchased from Invitrogen (Carlsbad, CA, USA). Primary antibodies against GAPDH, caspase-3, BAX, PARP, Bcl-2, Nrf2, GPX4, and SLC7A11 were purchased from CST (Danvers, MA, USA).

### 4.2. Plant Materials and Isolation of PXA

The strain was isolated from the fresh root of *Acanthus ilicifolius* L. collected from the Mangrove Nature Reserve in Guangxi, China. It was identified as *Diaporthe goulteri* by Sangon Biotech Co., Ltd., Shanghai, China. The ITS (GenBank ID: MW479162), TEF (GenBank ID: ON221692) and Tub (GenBank ID: ON221693) sequences of the strain were amplified by PCR to obtain 552 bp, 585 bp and 801 bp DNA fragments, respectively.

The fungal strain was cultured on slants of potato dextrose agar (PDA) at 25 °C for 5 days. Agar plugs were used to inoculate nine Erlenmeyer flasks (250 mL), each containing 100 mL of potato dextrose broth (PDB). Fermentation was carried out in 40 Erlenmeyer flasks (500 mL), each containing 70 g of rice. Distilled H_2_O (105 mL) was added to each flask, and the rice was soaked overnight before autoclaving at 120 °C for 30 min. After cooling to room temperature, each flask was inoculated with 5.0 mL of the seed culture containing mycelia and incubated at room temperature for 49 days. The culture was extracted thrice using EtOAc through cold maceration, and the organic solvent was evaporated to dryness under vacuum to afford a crude extract (7.6 g). This was subjected to silica gel column chromatography using cyclohexane-CH_3_OH (100:0 and 0:100, *v*/*v*) to afford a cyclohexane extract (1.4 g) and a CH_3_OH extract (5.6 g). The methanol extract was separated using octadecyl silica and eluted with MeOH-H_2_O (15:85, 45:55, 70:30, 100:0, *v*/*v*) to yield four fractions (w1–w4). Fraction w3 (2.1 g) was applied to a silica gel column and eluted with CH_2_Cl_2_-MeOH (30:1, 18:1, 9:1,4:1, 0:100, *v*/*v*) to obtain fractions (w3-1–w3-5). The w3-1 fraction was purified on a Sephadex LH-20 column, eluted with MeOH, and separated using high-performance liquid chromatography (HPLC) in 65% acetonitrile-35% water (0.1% formic acid) to yield PXA (t_R_ = 24.0 min, 3 mL/min, 41.1 mg, 0.54% yield).

### 4.3. Cell Culture and Transfection

The CRC cell lines HCT116 and HT29 were purchased from the cell bank of China Science Academy (Shanghai, China), and HCT116 cells were cultured in RPMI-1640 medium supplemented with 10% FBS, 0.5% penicillin, and streptomycin. HT29 cells were maintained in McCoy’s 5A medium with supplemented 10% FBS, 0.5% penicillin, and streptomycin. All cell lines were grown at 37 °C in a 5% CO_2_ atmosphere. The stable cell lines were established as we previously described [43]. HCT116 and HT29 cells were infected with a lentivirus targeting Nrf2 for 48 h and then incubated with a medium containing puromycin (3 μg/mL) to generate cells stably overexpressing Nrf2.

### 4.4. Cell Viability Assay

HCT116 and HT29 cells (5 × 10^3^ cells/well) were seeded in 96-well plates and incubated overnight. Then, cells were treated with indicated drugs at 37 °C for 48 h. Cell viability was determined by CCK-8 assay according to the instructions of the assay kit, and the absorbance of each well at 450 nm was measured using a microplate reader (Bio-Rad, Hercules, CA, USA).

### 4.5. Flow Cytometry Assay

The apoptosis assay was measured using Annexin V-FITC/PI double staining. HCT116 and HT29 cells were seeded (1 × 10^5^ cells/well) in six-well plates and treated with PXA and CDDP for 48 h, according to the manufacturer’s protocol. Cells were resuspended in 500 μL of 1× binding buffer containing 5 μL of Annexin V-FITC and incubated for 10 min in the dark. Then, 10 μL of PI work solution was added and incubated for another 5 min. The percentage of apoptotic cells was analyzed by flow cytometry (BD, San Diego, CA, USA).

Cell ROS analysis was conducted using an ROS Assay Kit. HCT116 and HT29 cells were treated with PXA and CDDP for 24 h; then, cells were incubated with a DCFH-DA probe (10 μM) at 37 °C for 20 min according to the instructions of the manufacturer. The ROS levels (DCF fluorescence) were observed and analyzed by fluorescence microscopy (Leica DMi8, Wetzlar, Germany) and flow cytometry (BD, San Diego, CA, USA).

### 4.6. ELISA

According to the manufacturer’s instructions, the levels of GSH, SOD and HO-1 in HCT116 and HT29 cells were measured using the ELISA kits.

### 4.7. Comet Assay

HCT116 and HT29 cells were treated with PXA and CDDP for 24 h. The DNA damage levels were detected using the Comet Assay Kit according to the instructions of the manufacturer [44].

### 4.8. Cellular Thermal Shift Assay

HCT116 and HT29 cells were cultured in a 10 cm dish and treated with PXA for 3 h. Subsequently, the cells were lysed using RIPA buffer supplemented with a 1% protease inhibitor cocktail. The resulting lysates were divided into six PCR tubes and subjected to a series of temperature treatments at 25, 46, 50, 54, 58, and 62 °C for 3 min. Following this, the samples underwent three freeze–thaw cycles in liquid nitrogen. After centrifugation at 12,000 rpm for 20 min at 4 °C, the supernatant was collected and subjected to analysis via Western blotting.

### 4.9. Liperfluo Staining

HCT116 and HT29 cells were treated with PXA and CDDP for 24 h. Cells were stained with Liperfluo (10 μM) for 30 min at 37 °C. The fluorescence of Liperfluo was observed using an inverted fluorescence microscope (Leica DMi8, Wetzlar, Germany).

### 4.10. Western Blot Analysis

Total protein was extracted using RIPA buffer mixed with a 1% protease inhibitor cocktail. Protein concentrations were quantified using a BCA kit. Equal amounts of protein samples were subjected to SDS-PAGE and subsequently transferred to 0.45 mm PVDF membranes (EMD Millipore, Bedford, MA, USA). The PVDF membranes were blocked in 5% skimmed milk for 2 h at room temperature, and then the membrane was further incubated with the primary antibody overnight at 4 °C. This was followed by incubation with the corresponding secondary antibody for 1 h at room temperature. Protein bands were visualized using Odyssey CLx Infrared Imaging System (Licro, NE, USA).

### 4.11. CRC Xenograft Zebrafish Model

Animal experiments in this study were approved by and performed according to the guidelines of the Animal Ethics Committee of Guangxi Medical University (authorization number: 202311002). The study involving AB wild-type zebrafish tumor model was established as we previously described [45]. Briefly, HCT116 cells were stained with the red, fluorescent dye CM-Dil solution for 30 min at 37 °C. Then, we injected CM-Dil-stained SKOV-3 cells into the zebrafish embryos at 2 days post-fertilization (2 dpf) using a microinjector (Nanoject III, Drummond, USA). After 3 dpf, the zebrafish were randomly divided into six groups to treat with the vehicle (DMSO), CDDP (3000 ng/mL), PXA (100 ng/mL, 1000 ng/mL, and 10,000 ng/mL), and CDDP (3000 ng/mL) + PXA (1000 ng/mL). After 48 h of treatment (at 5 dpf), fluorescence images of HCT116-CM-Dil cells in zebrafish were acquired using a fluorescence stereo-microscope (Olympus U-HGLGPSD, Tokyo, Japan).

### 4.12. Statistical Analysis

Data were statistically analyzed with SPSS software version 26.0 (SPSS Inc., Chicago, IL, USA). Differences between multiple groups were determined using one-way ANOVA, and data are presented as the mean ± SD with GraphPad Prism 6.0 (GraphPad Software, CA, USA). The Synergy scores of the two-drug combinations were acquired using the Synergy-finder software (https://synergyfinder.fimm.fi/, accessed on 7 June 2024), based on the Zero Interaction Potency model. Synergy scores > 10 are considered synergistic. A value of *p* < 0.05 indicated statistical significance.

## 5. Conclusions

In summary, our study demonstrates that PXA, a xanthone dimer compound isolated from the endophytic fungus *Diaporthe goulteri*, has potential as a novel Nrf2 pathway inhibitor. PXA can hypersensitize CRC cells to CDDP by targeting Nrf2 to induce ferroptosis, providing a promising chemosensitizing therapeutic strategy for treating cancer.

## Data Availability

The data that support the findings of this study are available from the corresponding author upon reasonable request.

## Supplementary Materials

The following supporting information can be downloaded at: https://www.mdpi.com/article/10.3390/md22080357/s1, Figure S1: UV spectrum of PXA; Figure S2: IR spectrum of PXA; Figure S3: ^1^H-NMR spectrum of PXA; Figure S4: ^13^C-NMR spectrum of PXA; Figure S5: HR-ESI-MS spectrum of PXA; Figure S6: The HPLC chromatographic analysis of PXA; Table S1: IC_50_ values of PXA on various cancer cells.
