# Supplementary material for: Identification of Penexanthone A as a Novel Chemosensitizer to Induce Ferroptosis by Targeting Nrf2 in Human Colorectal Cancer Cells
Source: Mar Drugs. 2024 Aug 6;22(8):357. doi: 10.3390/md22080357 (PMC11355289; doi:10.3390/md22080357)
Supplement: Supplementary file 1 [file marinedrugs-22-00357-s001.zip › SupplementaryMaterials_marinedrugs-3116505.pdf]

Supplementary Materials:

Article

Identification of Penexanthone A as a novel chemosensitizer to induce ferroptosis by targeting Nrf2 in human colorectal cancer cells

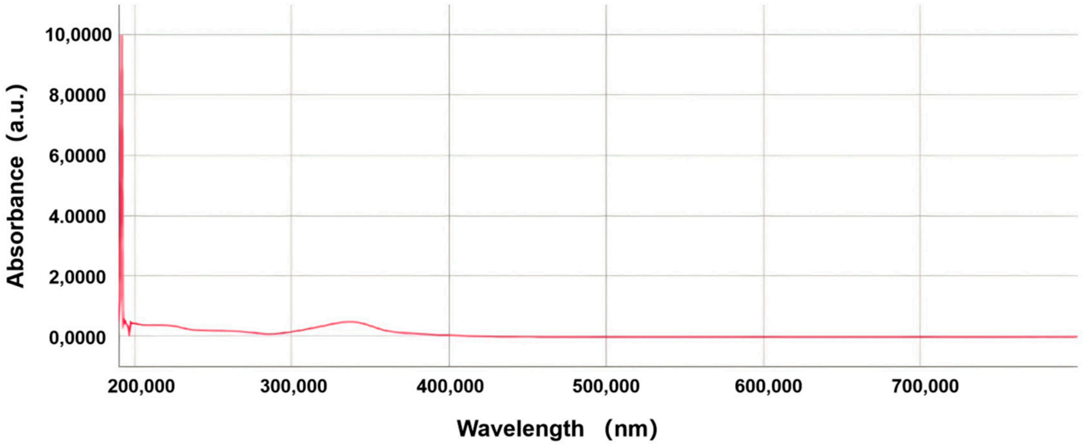

Figure S1. UV spectrum of PXA

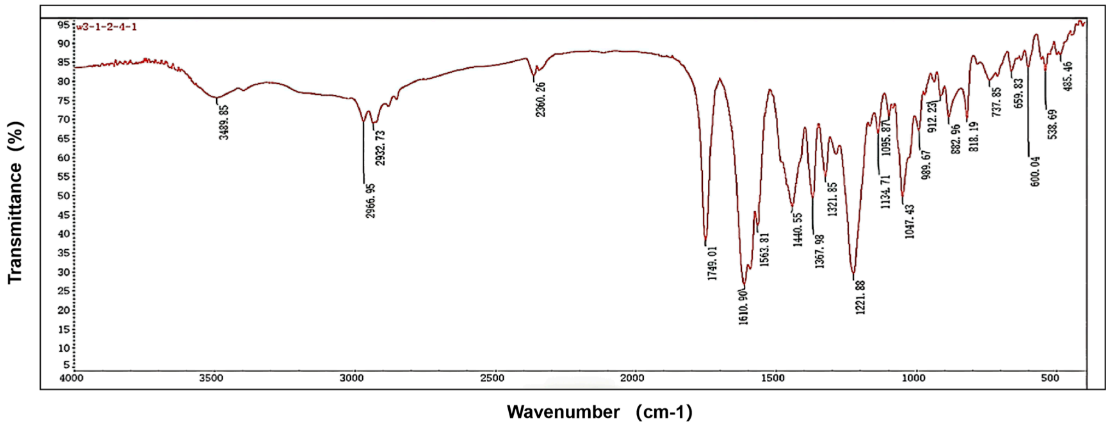

Figure S2. IR spectrum of PXA

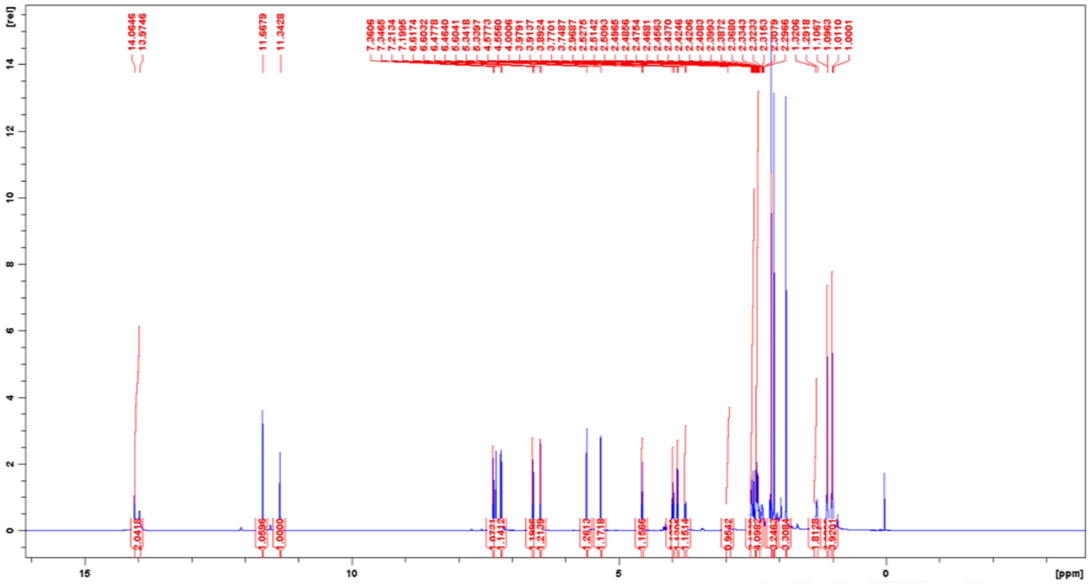

Figure S3 ^{1}H-NMR spectrum of PXA

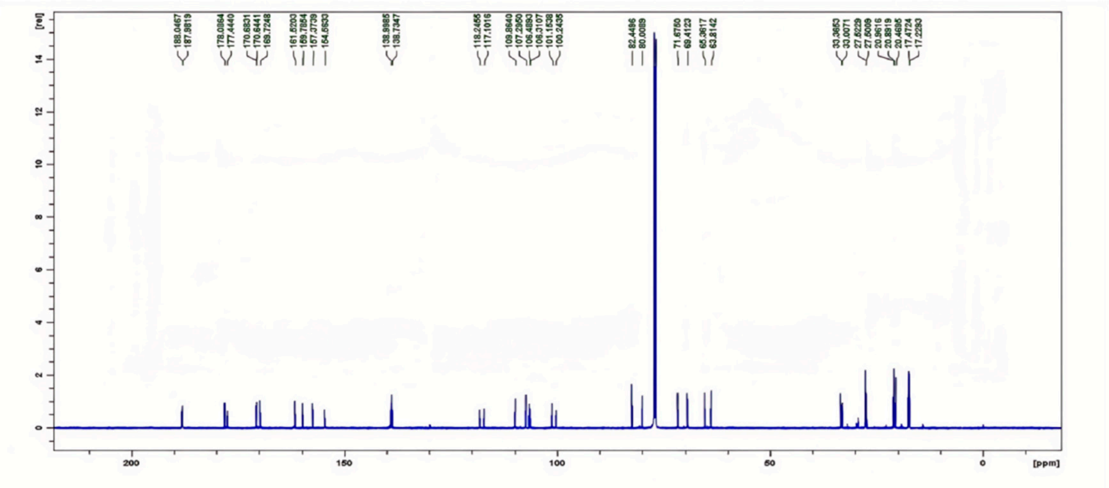

Figure S4 ^{13}C-NMR spectrum of PXA

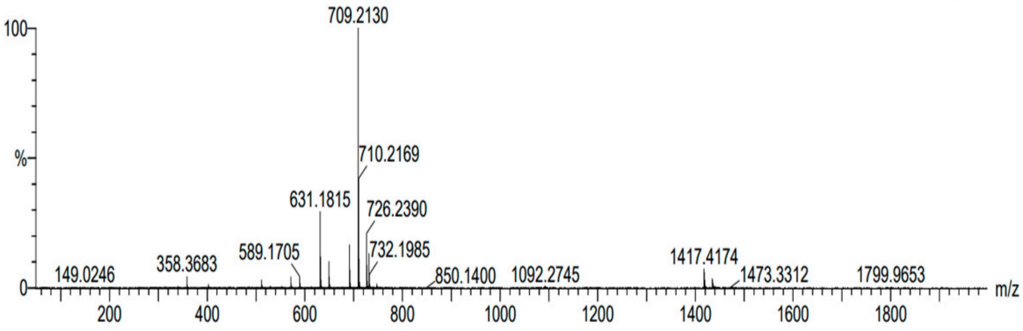

Figure S5 HR-ESI-MS spectrum of PXA

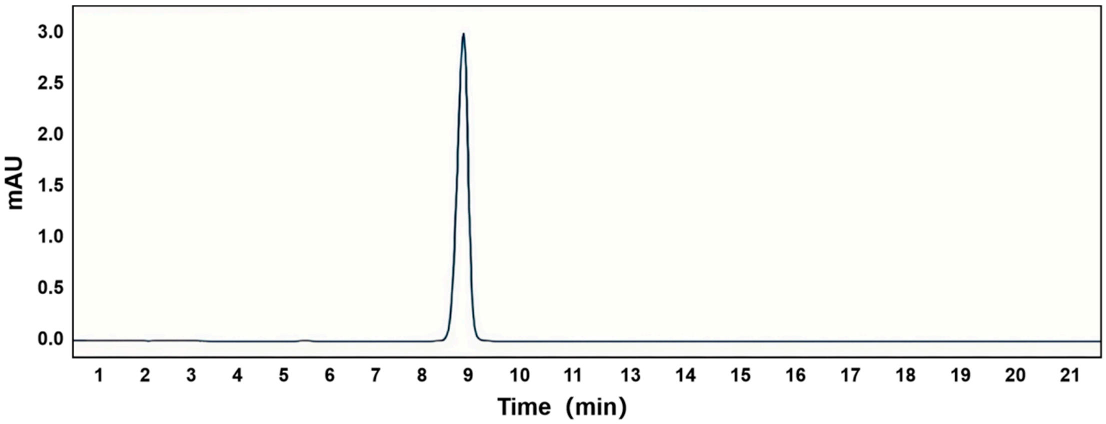

Figure S6. The HPLC chromatographic analysis of PXA.

Table S1. IC_{50} values of PXA on various cancer cells.

Compound	SKOV3	MCF-7	A549	HCT116	PC3
PXA (μM)	3.71	3.42	4.30	1.27	7.08
