# Supplementary material for: Identification of Penexanthone A as a Novel Chemosensitizer to Induce Ferroptosis by Targeting Nrf2 in Human Colorectal Cancer Cells
Source: Mar Drugs. 2024 Aug 6;22(8):357. doi: 10.3390/md22080357 (PMC11355289; doi:10.3390/md22080357)

Full unedited gel for Figure.1E

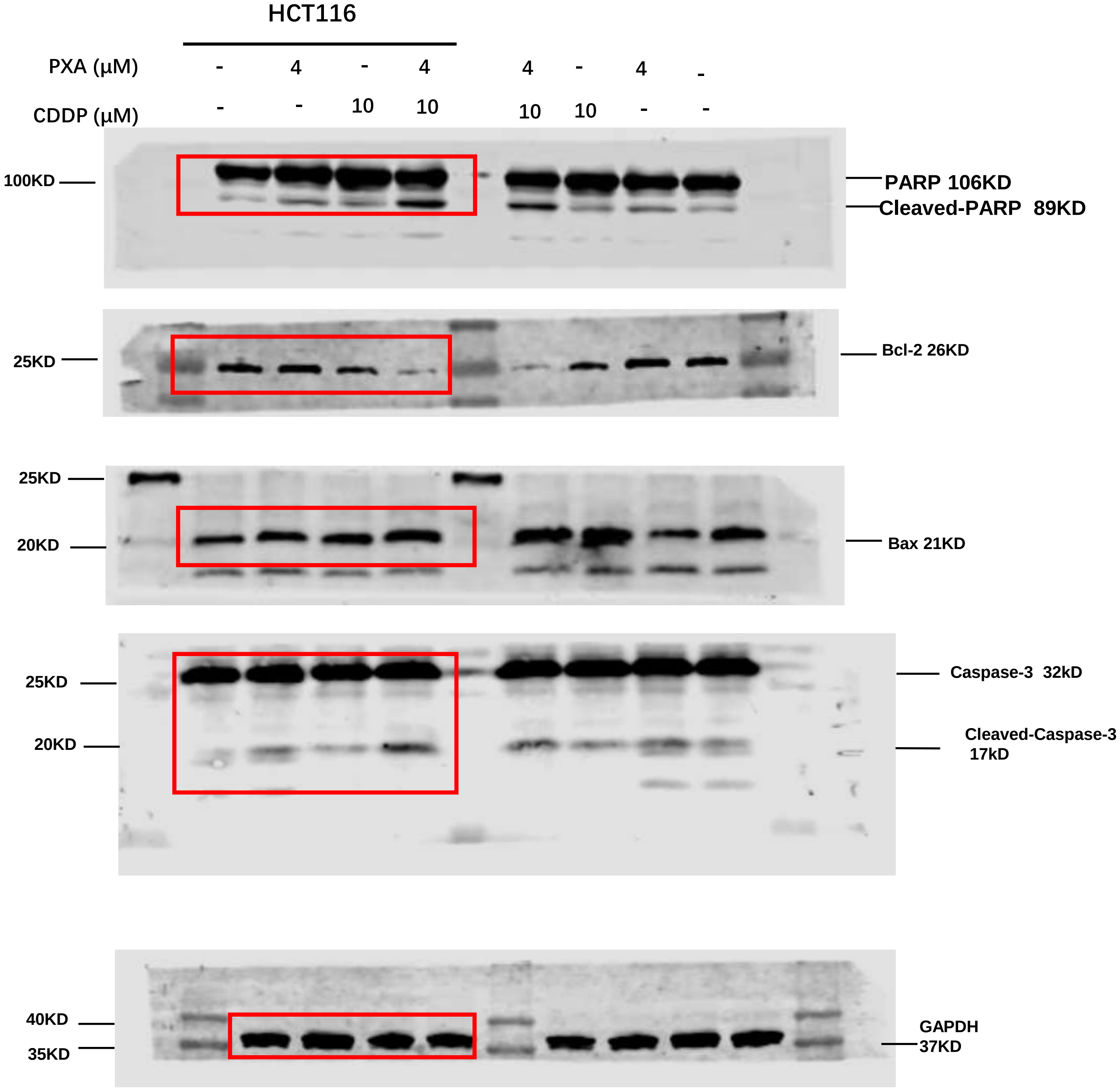

Full unedited gel for Figure.1E

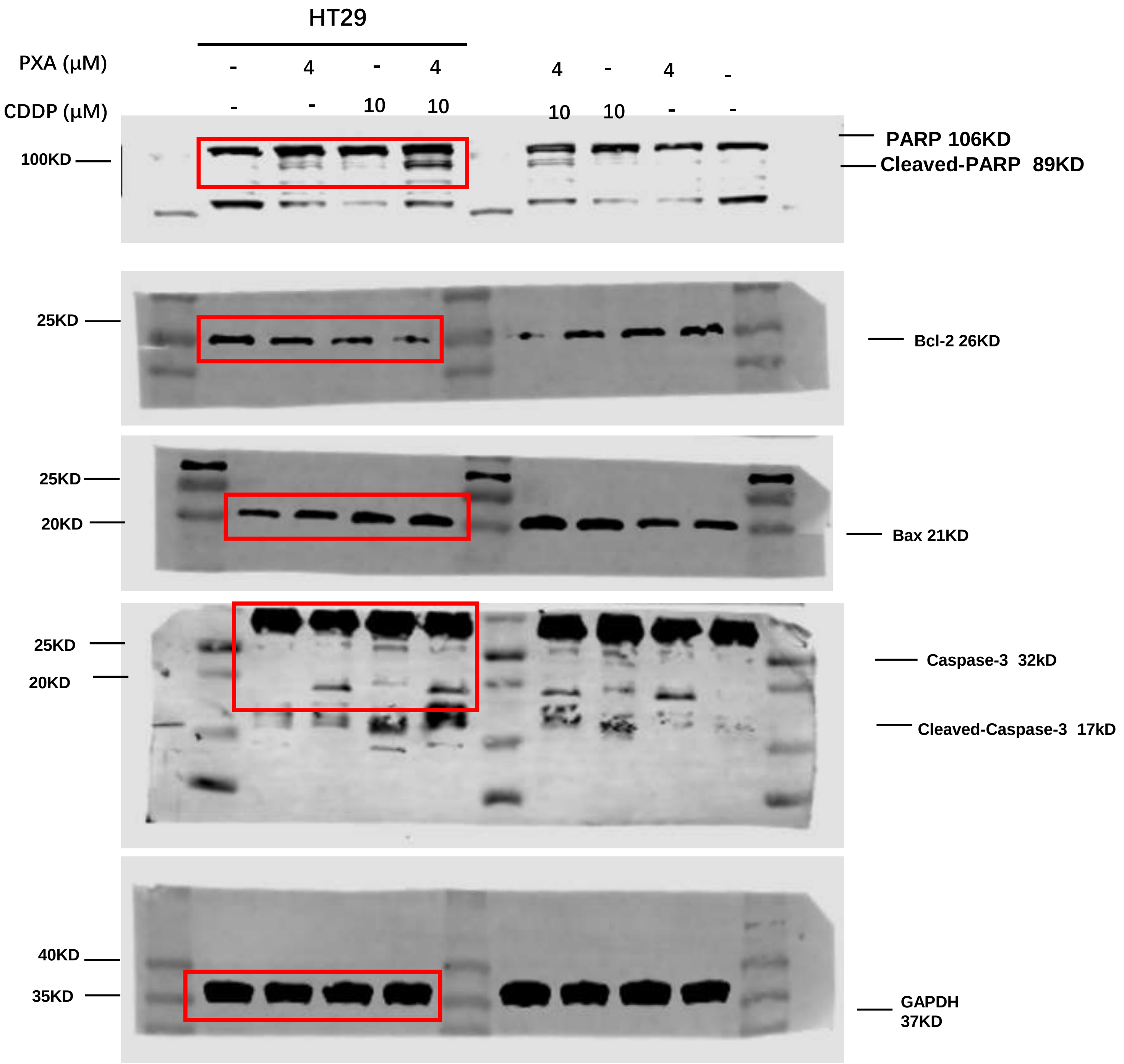

Full unedited gel for Figure.4A

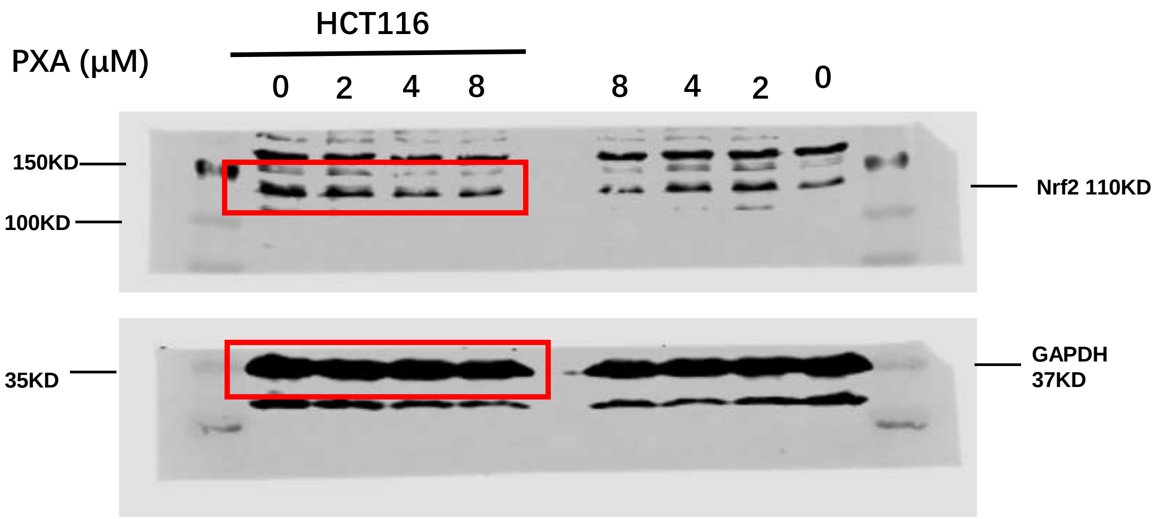

Full unedited gel for Figure.4B

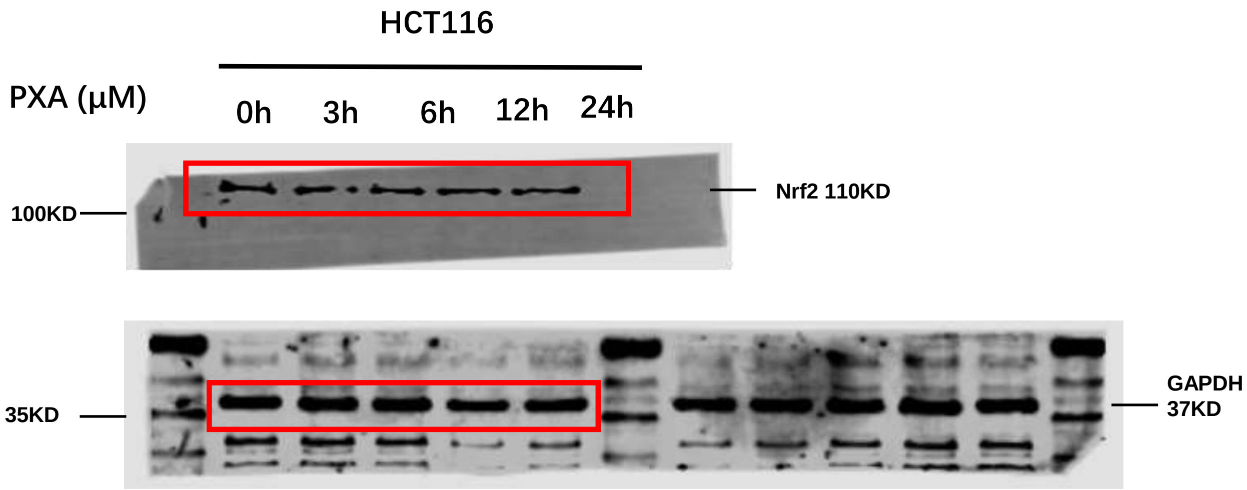

Full unedited gel for Figure.4A

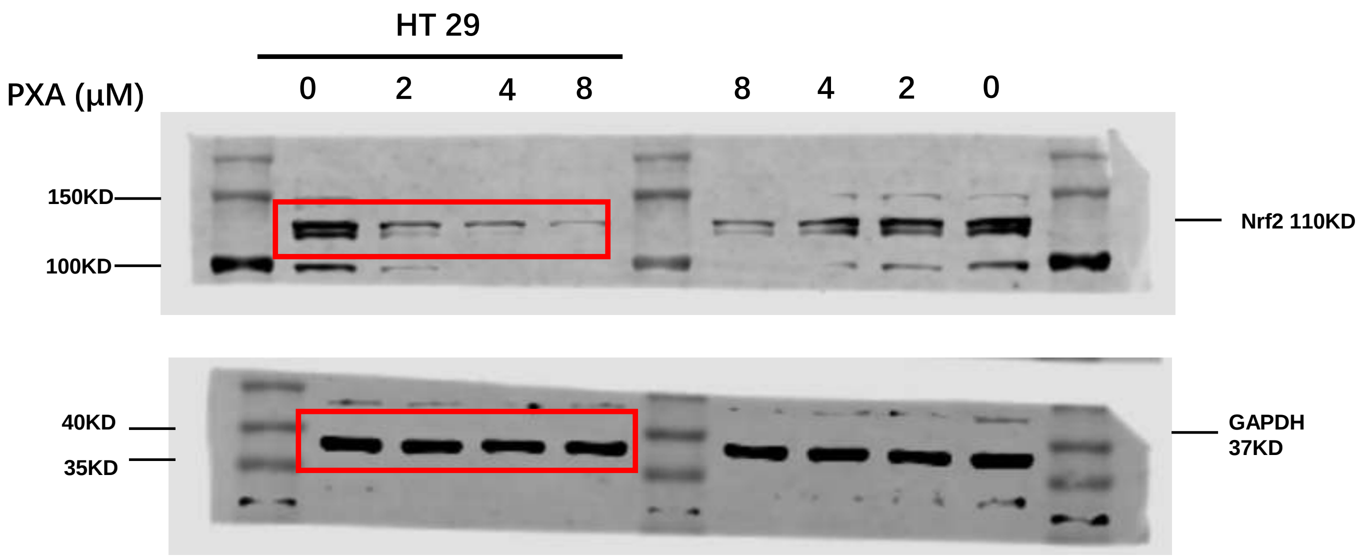

Full unedited gel for Figure.4B

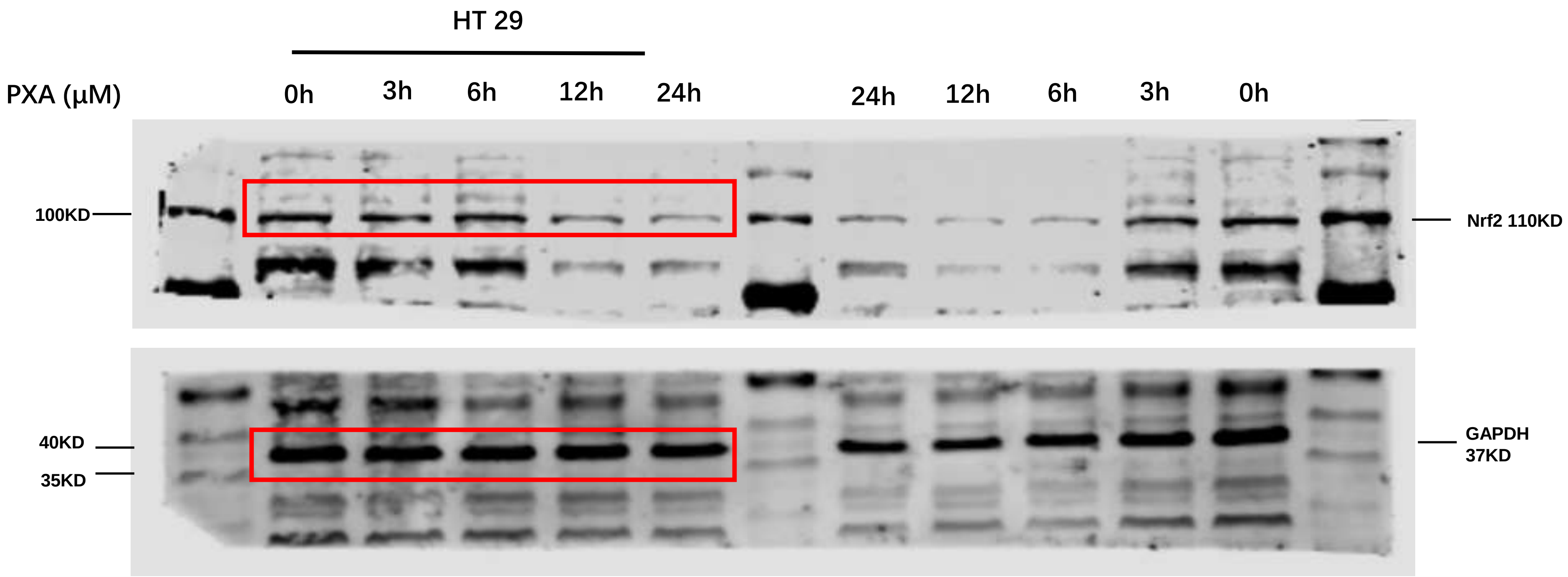

Full unedited gel for Figure.4C

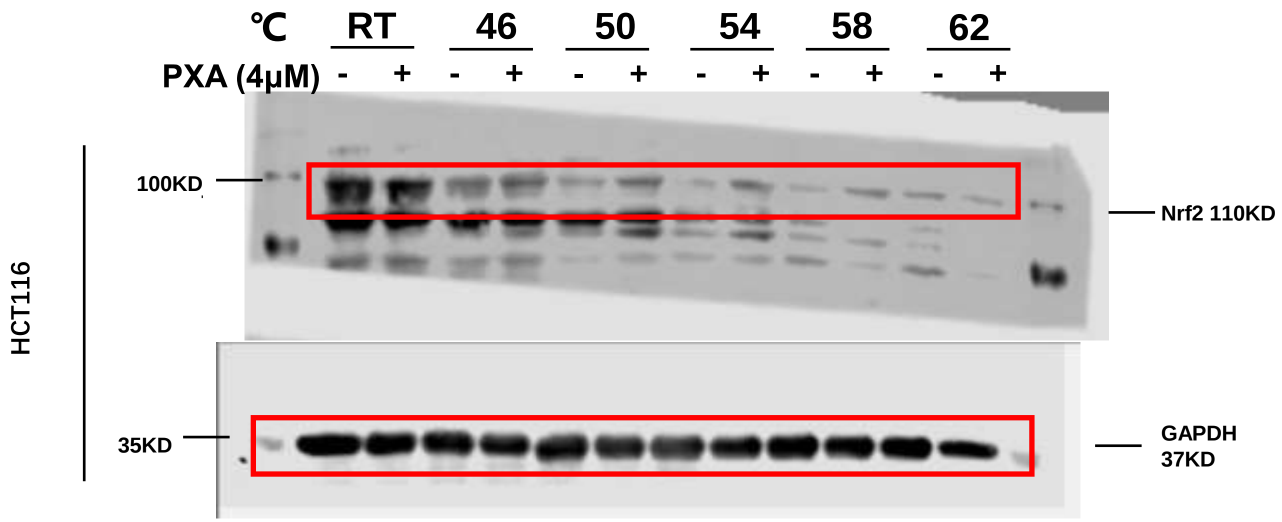

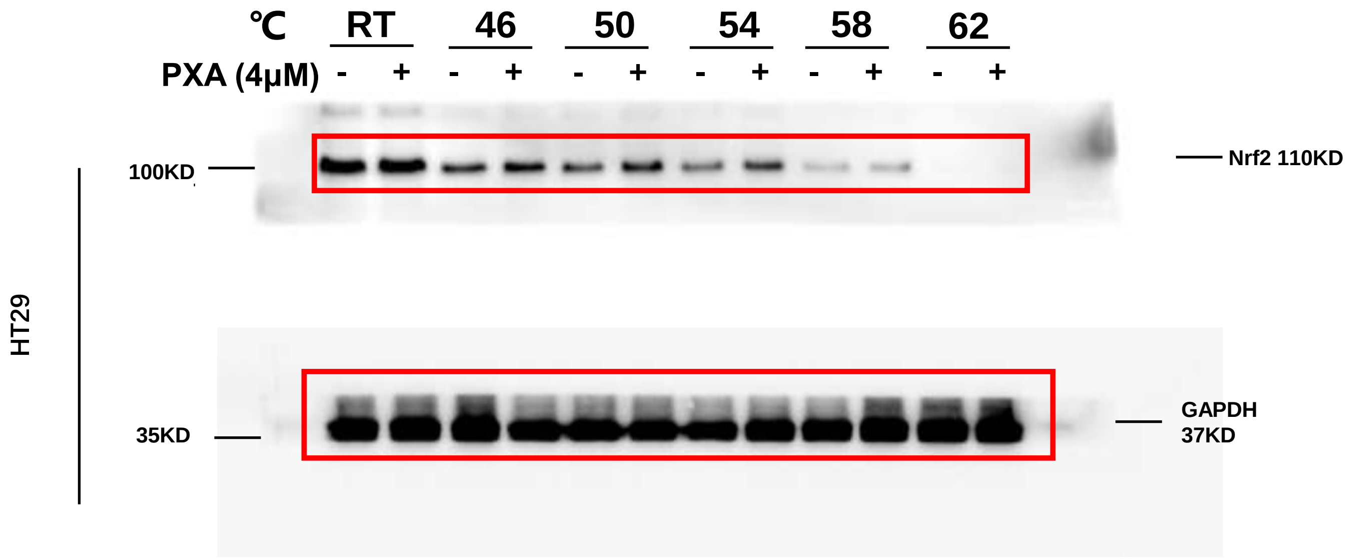

Full unedited gel for Figure.5A

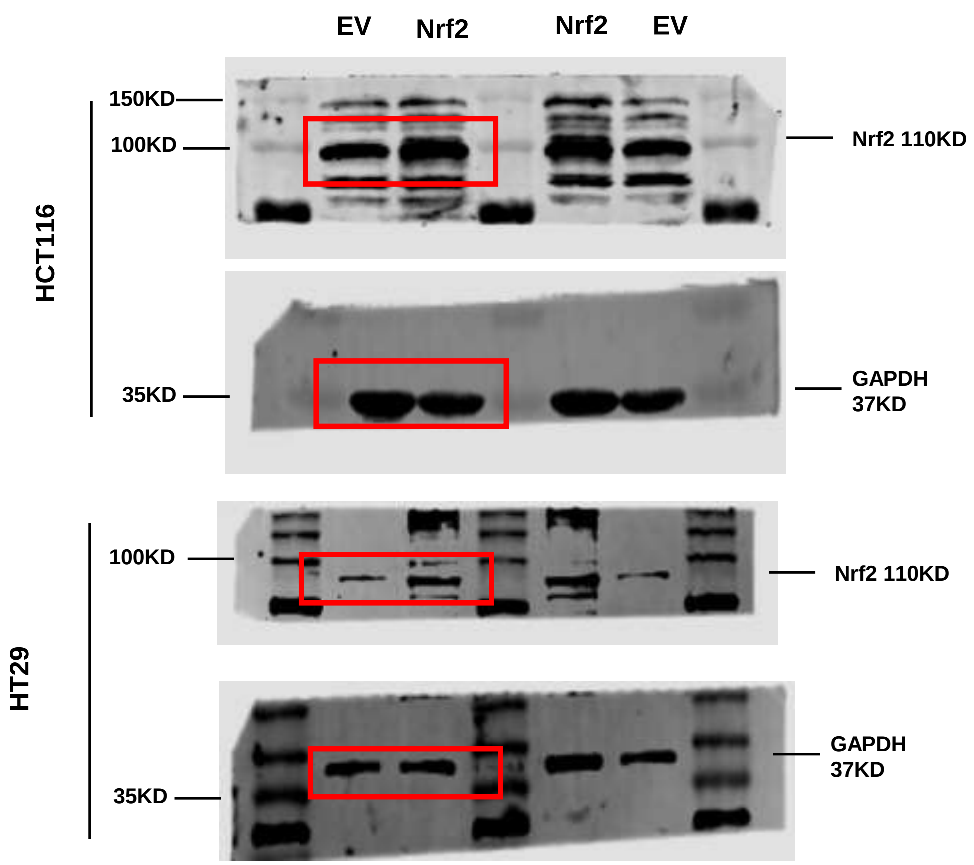

Full unedited gel for Figure.5B

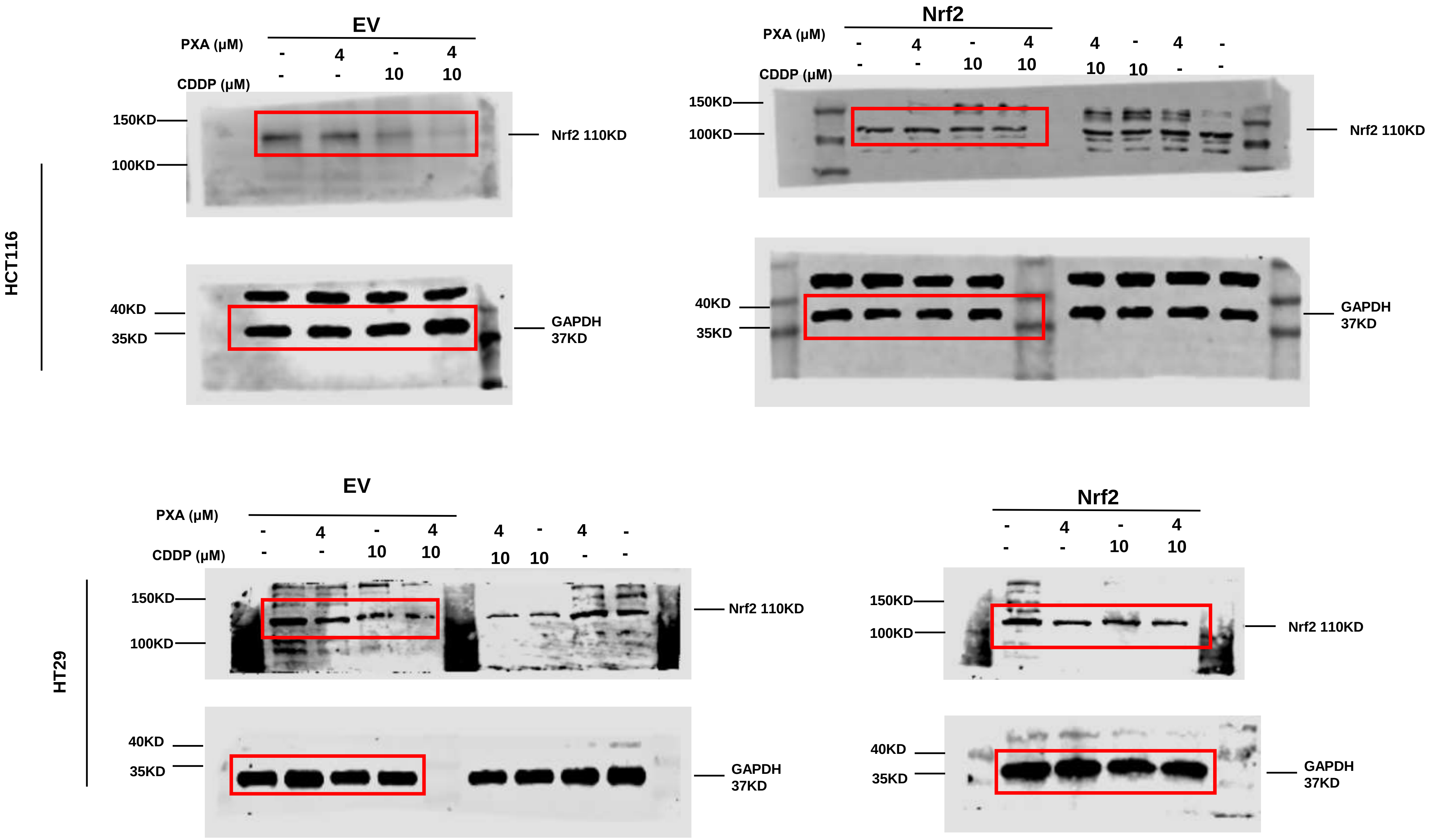

Full unedited gel for Figure.6C

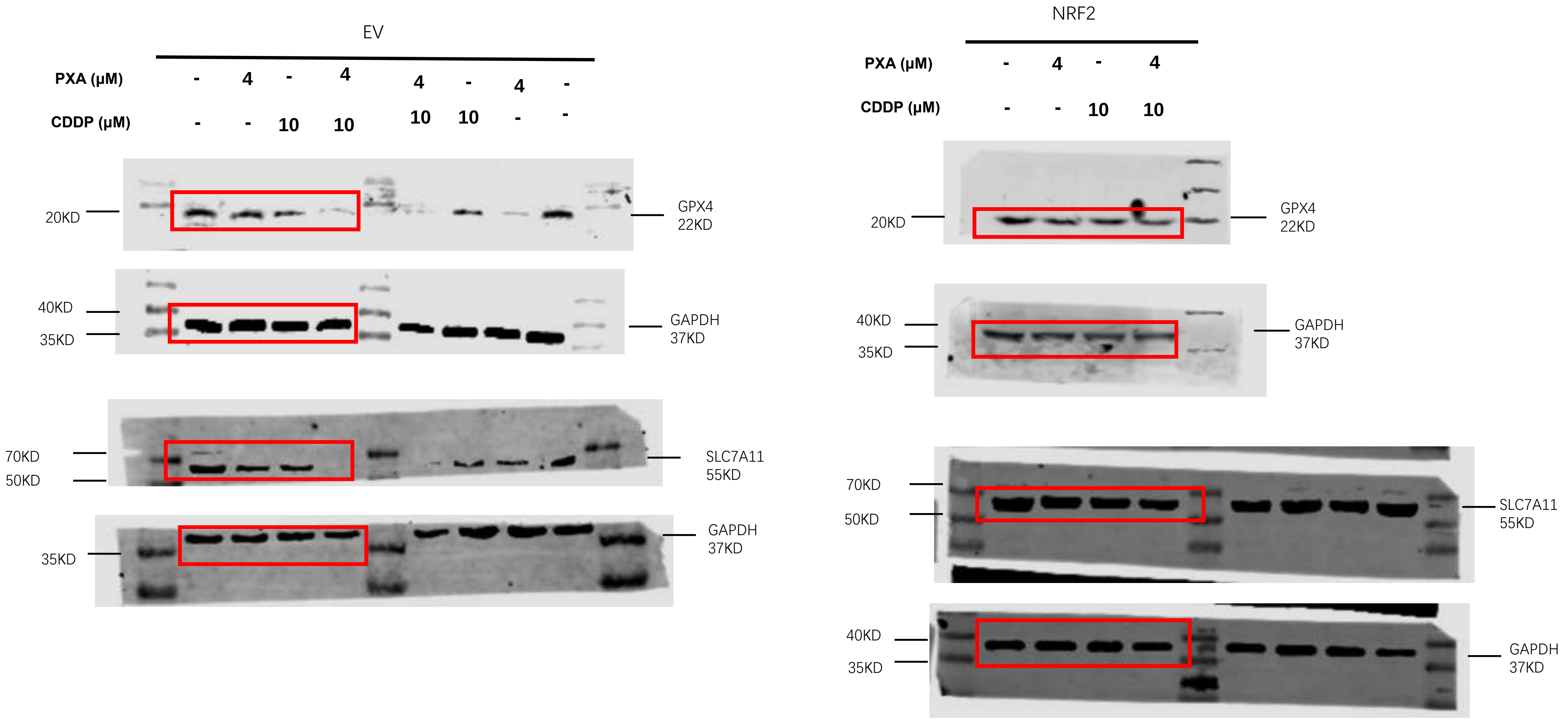

Full unedited gel for Figure.6C

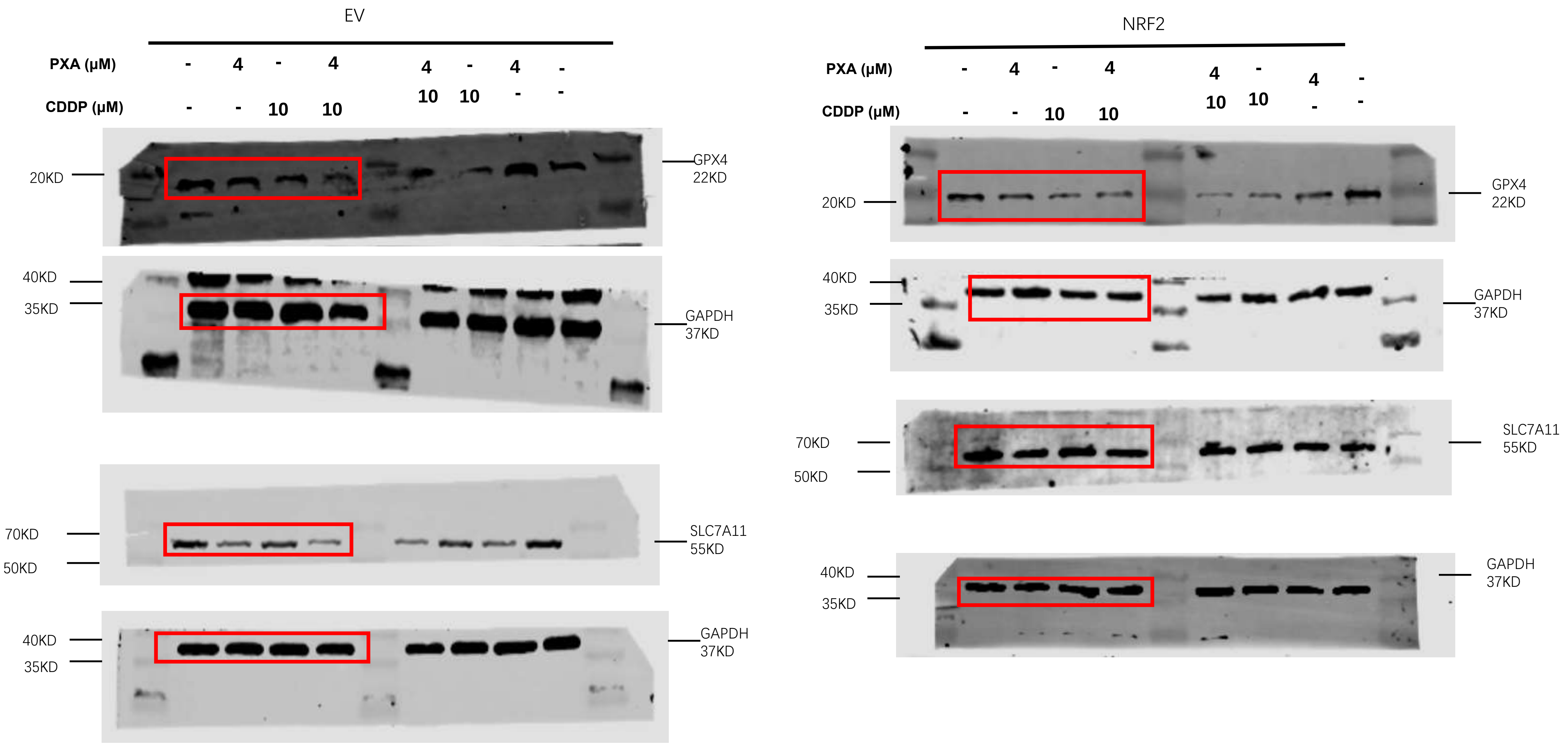

Supplement: Supplementary file 1 [file marinedrugs-22-00357-s001.zip › uncut gel blots_marinedrugs-3116505.pdf]
